# Therapeutic silencing miR-146b-5p improves cardiac remodeling in a porcine model of myocardial infarction by modulating the wound reparative phenotype

**DOI:** 10.1007/s13238-020-00750-6

**Published:** 2020-08-26

**Authors:** Yiteng Liao, Hao Li, Hao Cao, Yun Dong, Lei Gao, Zhongmin Liu, Junbo Ge, Hongming Zhu

**Affiliations:** 1 Research Center for Translational Medicine, Shanghai East Hospital, Tongji University School of Medicine, Shanghai, 200120 China; 2 Department of Cardiology, Shanghai Tenth People’s Hospital, Tongji University School of Medicine, Shanghai, 200072 China; 3 Department of Cardiovascular Surgery, Shanghai East Hospital, Tongji University School of Medicine, Shanghai, 200120 China; 4 Department of Ultrasound in Medicine, Shanghai East Hospital, Tongji University School of Medicine, Shanghai, 200120 China; 5 Department of Cardiology, Shanghai Institute of Cardiovascular Diseases, Zhongshan Hospital of Fudan University, Shanghai, 200032 China

**Keywords:** cardiac fibrosis, microRNA, porcine model, myocardial infarction

## Abstract

Fibrotic remodeling is an adverse consequence of immune response-driven phenotypic modulation of cardiac cells following myocardial infarction (MI). MicroRNA-146b (miR-146b) is an active regulator of immunomodulation, but its function in the cardiac inflammatory cascade and its clinical implication in fibrotic remodeling following MI remain largely unknown. Herein, miR-146b-5p was found to be upregulated in the infarcted myocardium of mice and the serum of myocardial ischemia patients. Gain- and loss-of-function experiments demonstrated that miR-146b-5p was a hypoxia-induced regulator that governed the pro-fibrotic phenotype transition of cardiac cells. Overexpression of miR-146b-5p activated fibroblast proliferation, migration, and fibroblast-to-myofibroblast transition, impaired endothelial cell function and stress survival, and disturbed macrophage paracrine signaling. Interestingly, the opposite effects were observed when miR-146b-5p expression was inhibited. Luciferase assays and rescue studies demonstrated that the miR-146b-5p target genes mediating the above phenotypic modulations included *interleukin 1 receptor associated kinase 1* (*IRAK1*) and *carcinoembryonic antigen related cell adhesion molecule 1* (*CEACAM1*). Local delivery of a miR-146b-5p antagomir significantly reduced fibrosis and cell death, and upregulated capillary and reparative macrophages in the infarcted myocardium to restore cardiac remodeling and function in both mouse and porcine MI models. Local inhibition of miR-146b-5p may represent a novel therapeutic approach to treat cardiac fibrotic remodeling and dysfunction following MI.

**Electronic supplementary material:**

The online version of this article (10.1007/s13238-020-00750-6) contains supplementary material, which is available to authorized users.

## Introduction

Myocardial infarction is the leading cause of mortality and morbidity worldwide (Nagpal et al., 2016), indicating an urgent need for studies investigating the underlying mechanisms of MI to identify innovative therapeutic strategies.

Pathological cardiac remodeling is characterized by complex multicellular alterations, such as cardiomyocyte death, immune cell activation, and excessive deposition of the extracellular matrix, that exacerbate cardiac dysfunction, and often progresses to heart failure (Sutton and Sharpe, 2000; Prabhu and Frangogiannis, 2016a, b; Shiraishi et al., 2016). Cells are well known to sense their surrounding physical and signaling environments and respond accordingly by altering their functions and fates (Zhu et al., 2014a, 4b). Pioneering studies have demonstrated that adverse remodeling following MI is caused by the phenotypic modulation of cardiac cells, with the inappropriate and untimely activation and resolution of inflammation being a crucial driving factor (Epelman et al., 2015; Westman et al., 2016; Meyer et al., 2017; Huang and Frangogiannis, 2018). Thus, therapeutic modulation of immunoregulatory factors and reparative phenotypes of resident cells may be a promising approach for preventing post-infarction remodeling (Prabhu and Frangogiannis, 2016a, b).

MicroRNAs are functional, single-stranded, short noncoding RNAs that regulate a large array of biological processes through the degradation or inhibition of target messenger RNAs (mRNAs). The miR-146 family was the first family of microRNAs reported to be involved in mammalian immunomodulation (Taganov et al., 2006; Baltimore et al., 2008). However, the functions of these microRNAs in the cardiac inflammatory cascade and adverse remodeling remain unclear.

To address this, we generated two animal models (mouse and pig) to demonstrate the benefits of inhibiting miR-146b-5p post-MI. The results indicated that hypoxia induced miR-146b-5p expression through the NF-κB signaling pathway. miR-146b-5p gain-of-function was found to activate fibroblast proliferation, migration, and fibroblast to myofibroblast transition (FMT), impair endothelial cell function and stress survival, and disrupt macrophage paracrine signaling. The opposite effects were observed upon miR-146b-5p loss-of-function. *IRAK1* and *CEACAM1* were found to be the targets of miR-146b-5p in the above phenotypic modulations. The local delivery of a miR-146b-5p antagomir significantly reduced fibrosis and cell death, and upregulated capillary and reparative macrophages in infarcted myocardium to restore cardiac remodeling and function in both mice and porcine MI models.

## Results

### miR-146b-5p was upregulated in patients with chronic total occlusion (CTO) and in a murine MI model

**Figure 1:**
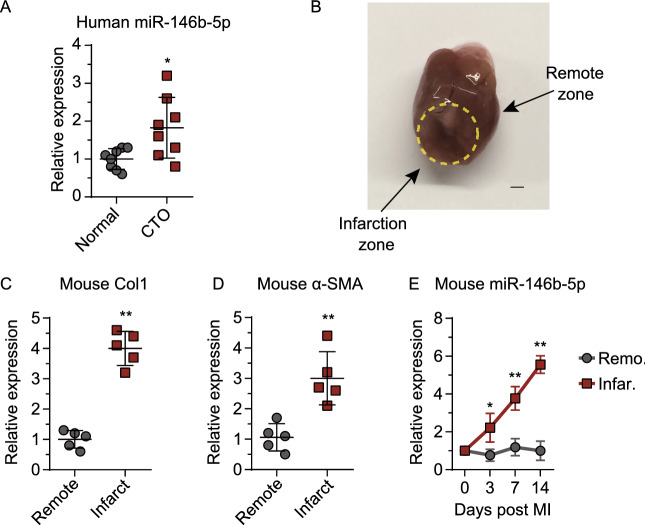
**miR-146b-5p is upregulated after myocardial infarction**. (A) Levels of miR-146b-5p in the plasma of patients with chronic total occlusion (CTO) (*n* = 8 per group). (B) Representative images of infarcted mouse heart. Myocardium samples from different anatomic locations were harvested for the following analysis. (C and D) Expression levels of fibrotic genes *Col1* (*COL1A1*) and *αSMA* (*ACTA2*) in myocardium samples (3 hearts, 5 samples per heart). (E) Expression levels of miR-146b-5p in mouse myocardium from different locations and time points, as measured by qPCR (*n* = 5 per group). Data are expressed as the mean ± SD. *P < 0.05 vs. control; **P < 0.01 vs. control. Data in panel (A), (C), and (D) were analyzed using Student’s unpaired *t*-test. Data in panel (E) was analyzed using one-way ANOVA followed by Tukey’s post-hoc analysis. Remo, remote zone; Infar, infarction zone.

CTO consists of the complete obstruction of a coronary artery, resulting in severe myocardial ischemia. Using quantitative real-time polymerase chain reaction (qPCR), we found that miR-146b-5p expression was significantly increased in the plasma of CTO patients compared to no-ischemia volunteers (*n* = 8 per group, *P* = 0.02; Fig. 1A). Clinical parameters and medical histories were available for all patients (Table S1). To elucidate the connection between plasma miR-146b-5p expression and myocardial ischemia, a mouse MI model was constructed, and qPCR was performed. Morphological observation and expression of the fibrotic genes *COL1A1* (*Col1*) and *ACTA2* (*αSMA*) were used to validate myocardial ischemia (Fig. 1B–D). We found that miR-146b-5p expression increased continuously in the infarct zone (reaching approximately 5-fold above the baseline), but not in the remote myocardium, over 14 days following the induction of MI (Fig. 1E).

### Hypoxia upregulated miR-146b-5p in fibroblasts, endothelial cells, and macrophages may through NF-κB signaling

Given that miR-146b-5p is upregulated in the ischemic myocardium, we investigated which cell types expressed miR-146b-5p under ischemia and the mechanism by which it is induced.

**Figure 2:**
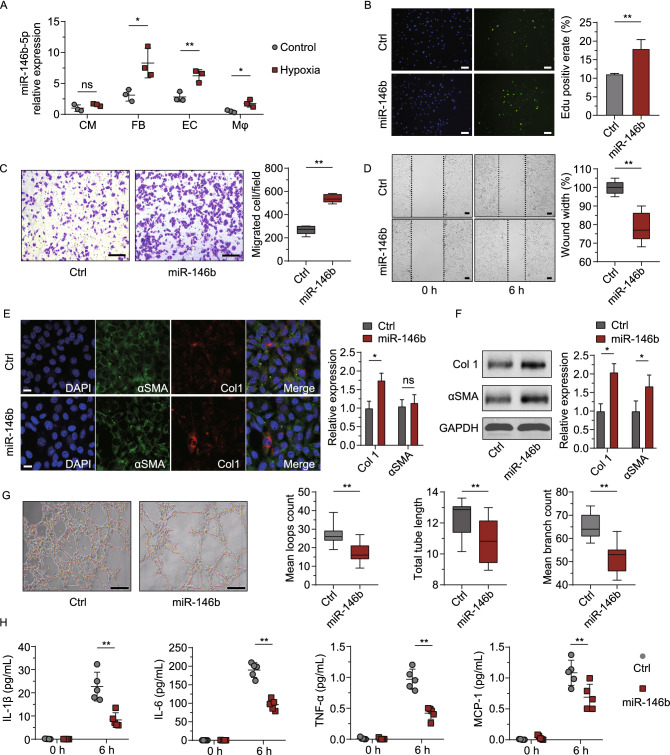
**Hypoxia-induced miR-146b-5p expression acts as a phenotypic modulator in fibroblasts, endothelial cells, and macrophages.** (A) The expression profile of miR-146b-5p in primary cultured cardiac cells under hypoxia was studied using qPCR (*n* = 3 per group, 3 replicates). Multiple phenotypic studies were performed after 24 h of treatment with the miR-146b-5p mimic (miR-146b) or control (Ctrl) (B–H). (B) Representative images for EdU staining and EdU^+^ cell quantification of cardiac fibroblasts (*n* = 7 samples per group, 5 random fields per sample). Bar = 200 μm. (C and D), Representative migration images and quantification of data from Transwell and scratch assays in mouse fibroblasts (NIH3T3, *n* = 5 per group, 5 random fields per sample). Bar = 200 μm. (E) Representative images for immunostaining and quantification of expression of the fibroblast-to-myofibroblast transition genes *Col1* (*COL1A1*) and *αSMA* (*ACTA2*) in cardiac fibroblasts (*n* = 3 per group, 7 fields per sample); bar = 5 μm. (F) Western blot analysis of the FMT genes in cardiac fibroblasts after treatment (*n* = 5). (G) Tube-formation assays of cardiac microvascular endothelial cells (CMVECs), with representative images on the left and quantification data on the right (*n* = 3 samples per group, 5 random fields per samples, 3 replicates). Bar = 200 μm. (H**)** ELISA of macrophage-secreted inflammatory factors (*n* = 5 per group). Data are expressed as the mean ± SD for at least three independent assays, unless otherwise noted. *P < 0.05; **P < 0.01; ns, not significant. Data were analyzed using Student’s *t*-test. CM, cardiomyocyte; FB, fibroblast; EC, endothelial cell; Mø, macrophage; IL, interleukin; TNF, tumor necrosis factor; MCP, monocyte chemotactic protein.

We utilized a classic *in vitro* hypoxia model to expose the major resident cell types of the mouse heart, including cardiomyocytes, fibroblasts, endothelial cells, and macrophages, to ischemic conditions. After treatment with 1% oxygen for 24 h, miR-146b-5p expression was significantly induced in fibroblasts, endothelial cells, and macrophages, with the fibroblasts and macrophages showing the largest increases in expression (2.7- and 3.5-fold, *P* = 0.03 for both) (Fig. 2A). Hypoxia treatment failed to induce miR-146b-5p expression in cardiomyocytes (*P* = 0.18). Additionally, hypoxic fibroblasts exhibited considerably higher expression of miR-146b-5p compared to the normoxic control (Fig. S1A), which is consistent with our ischemic myocardium data (Fig. 1E).

Transforming growth factor-β (TGF-β) signaling is well known as one of the primary mechanisms mediating cardiac remodeling following ischemia/hypoxia. Therefore, we investigated whether miR-146b-5p expression was activated by TGF-β signaling. As shown in Fig. S1B, treatment with 10 ng/mL TGF-β for 24 h greatly increased the expression of the fibrotic marker *αSMA* (about 2-fold, *P* = 0.01) in mouse fibroblasts; however, this treatment failed to induce miR-146b-5p expression (*P* = 0.66). Additionally, a luciferase reporter-based promoter activity assay indicated that the NF-κB promoter was activated during the 24-h hypoxic period (Fig. S1C). Using JSH-23, an NF-κB transcriptional inhibitor (30 μmol/L, 24-h treatment), we demonstrated that the inhibition of NF-κB transcriptional activity significantly reduced the expression of miR-146b-5p in fibroblasts (*P* < 0.01, Fig. S1D), endothelial cells and cardiac macrophages (both *P* < 0.01, Fig. S1E) under hypoxic conditions, suggesting that hypoxia-induced miR-146b-5p expression is at least partially mediated through NF-κB signaling. Collectively, these findings demonstrate that miR-146b-5p is characteristically induced in fibroblasts, endothelial cells, and macrophages by hypoxia. NF-κB signaling rather than TGF-β signaling involve in hypoxia-induced upregulation of miR-146b-5p in fibroblast.

### miR-146b-5p drove the pro-fibrotic transition of fibroblasts, impaired endothelial cell function and stress survival, and disrupted macrophage paracrine signaling

**Figure 3:**
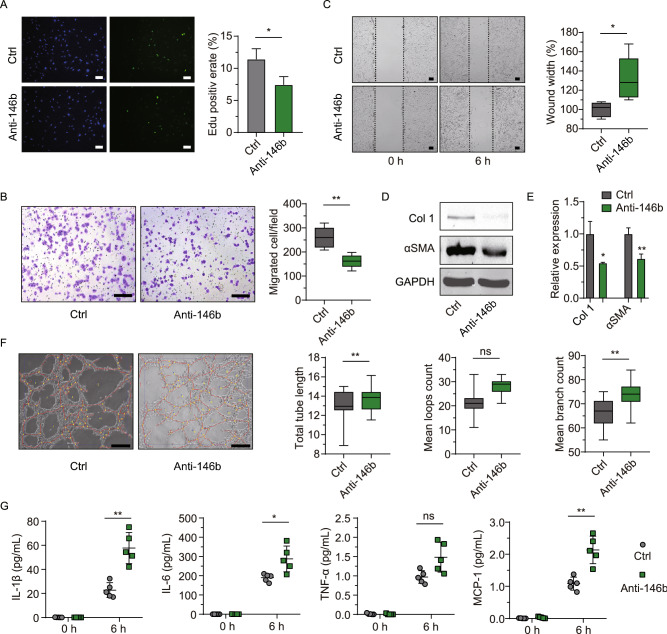
**Inhibition of miR-146b-5p consolidates the reparative phenotypes of fibroblasts, endothelial cells, and macrophages.** Multiple phenotypic studies were performed after 24 h of treatment with miR-146b-5p inhibitor (Anti-146b) or control (Ctrl). (A) Representative images for EdU staining and EdU^+^ cell quantification of cardiac fibroblasts (*n* = 7 samples per group, 5 random fields per sample). (B and C), Representative migration images and quantification, according to Transwell and scratch assays of mouse fibroblasts (NIH3T3, *n* = 5 per group, 5 random fields per sample). (D and E) Representative western blot images and quantification of expression of the fibroblast-to-myofibroblast transition genes *Col1* (*COL1A1*) and *αSMA* (*ACTA2*) in cardiac fibroblasts (*n* = 5 per group). (F) Tube-formation assays of cardiac microvascular endothelial cells (CMVECs), with representative images on the left and quantification data on the right (*n* = 3 samples per group, 5 random fields per samples, 3 replicates). (G) ELISA of macrophage-secreted inflammatory factors (*n* = 5 per group). Data are expressed as the mean ± SD for at least three independent assays unless otherwise noted. *P < 0.05 vs. control; **P < 0.01 vs. control; ns, not significant. Data were analyzed using Student’s *t*-test. IL, interleukin; TNF, tumor necrosis factor; MCP, monocyte chemotactic protein.

Pro-fibrotic myofibroblasts are derived from the FMT and are the main source of collagen and other ECM proteins during pathological fibrotic remodeling. To determine if miR-146b-5p regulates FMT, mouse fibroblasts were treated with miR-146b-5p mimic, miR-146b-5p inhibitor, and controls. The results of the gain-of-function experiments are shown in Fig. 2, and those of the loss-of-function experiments are provided in Fig. 3. We first performed a dose-response study in fibroblasts based on fibrotic gene expression (*COL1A1*) and found treatment with 20 nmol mimic to be a desirable concentration in regards to both economy and efficiency (Fig. S1F). Next, the pro-fibrotic phenotype was assessed in terms of proliferation, migration, and expression of FMT markers, as shown in Figs. 2B–F and 3A–E. Overexpression of miR-146b-5p markedly enhanced fibroblast proliferation by 1.6-fold (*P* = 0.01, Fig. 2B), as determined by EdU staining, while miR-146b-5p inhibition significantly reduced cell proliferation (*P* = 0.03, Fig. 3A). The Transwell assay indicated that the miR-146b-5p mimic increased fibroblast migration by 2-fold (*P* = 0.0001, Fig. 2C). Inhibition of miR-146b-5p significantly attenuated cell migration by up to 40% (*P* = 0.003, Fig. 3B). Similar trends were observed in the scratch assay (Figs. 2D and 3C). Furthermore, as shown in Fig. 2E, immunofluorescence staining revealed that the miR-146b-5p mimic increased the expression of the FMT markers *COL1A1* (*Col1*) and *ACTA2* (*αSMA*) by 1.8-fold and 1.1-fold (*P* = 0.02 and *P* = 0.84), respectively. Additionally, western blotting showed a 1.9-fold and 1.6-fold increase in Col1 and αSMA expression, respectively, after mimic treatment (Fig. 2F). As expected, the miR-146b-5p inhibitor significantly reduced Col1 and αSMA protein expression by 62% and 29%, respectively (*P* = 0.02 and *P* = 0.01; Fig. 3D and 3E).

Primary cardiac microvascular endothelial cells (CMVECs) were cultured and revealed that both overexpression and inhibition of miR-146b-5p regulated tube formation in CMVECs (Figs. 2G and 3F) and survival under starvation (Fig. S2). Upregulation of miR-146b-5p enhanced malnutrition-induced death in CMVECs and inhibited tube formation in Matrigel. Loop count, tube length, and branch count were significantly decreased after treatment with the miR-146b-5p mimic (Fig. 2G), whereas these were partially increased by inhibition of miR-146b-5p (*P* = 0.002 for loops and *P* = 0.0002 for branch counts; Fig. 3F).

Cardiac macrophages can regulate inflammation, angiogenesis, and tissue repair after MI by secreting factors and undergoing polarization (Nahrendorf and Swirski 2013; Swirski and Nahrendorf, 2013; Ma et al., 2018; Dick et al., 2019). In the present study, using resident macrophages isolated from MI hearts revealed that miR-146b-5p overexpression significantly decreased secretion of interleukin-1β (IL-1β), IL-6, tumor necrosis factor-α (TNF-α), and monocyte chemotactic protein 1 (MCP-1) (Fig. 2H; *P* = 0.002, 0.0001, 0.0002 and 0.02, respectively). However, miR-146b-5p inhibition strongly increased the amount of IL-1β, IL-6, and MCP-1 released into the culture supernatant (Fig. 3G). A growing body of evidence has demonstrated that early inflammatory signals set the stage for cardiac repair in the infarcted heart (Frangogiannis, 2012, 2014), suggesting that inflammatory secretion regulated by miR-146b-5p may influence endogenous repair following MI.

We also investigated the effect of miR-146b-5p inhibition on cardiomyocyte viability and found that miR-146b-5p inhibition did not have a significant effect on cardiomyocyte viability *in vitro* for up to 72 h (Fig. S3).

Collectively, these results demonstrate that miR-146b-5p regulates the pro-fibrotic phenotype and FMT of fibroblasts, tube formation, starvation survival of CMVECs, and inflammatory factor secretion by macrophages, indicating an important regulatory role for miR-146b-5p in the phenotypic modulation of cardiac cells following MI.

### miR-146b-5p may modulate cardiac cell phenotypes by targeting *IRAK1* and *CEACAM1*

In the present study, miR-146b-5p was found to directly bind to the 3′UTRs of *IRAK1* and *CEACAM1* based on luciferase reporter assays (Fig. S4A and S4B). *IRAK1* is a major downstream effector of IL-1β signaling. *CEACAM1* is a cell-cell adhesion molecule involved in differentiation, angiogenesis, apoptosis, and the innate and adaptive immune responses (Horst, 2006; Huang et al., 2015). Previously, little was known regarding the pathophysiological roles of *IRAK1* and *CEACAM1* in the heart. Interestingly, we found that miR-146b-5p exhibits preferential targeting in cardiac cells. In protein level, miR-146b-5p was more likely to inhibit *IRAK1* expression in fibroblasts and macrophages, whereas it tended to inhibit *CEACAM1* expression in endothelial cells (Fig. S4C–E). The *IRAK1* overexpression was able to significantly rescue miR-146b-induced phenotypic changes in fibroblasts and macrophages (Fig. S4F and S4G). Additionally, treatment with the miR-146b-5p mimic reduced endothelial cell survival by 66.4% under starvation, and decreased tube length, loop count, and branch count in tube formation assays (23.1%, 48.7%, and 49.3%, respectively). Lentivirus-mediated upregulation of *CEACAM1* significantly rescued miR-146b-induced endothelial cell survival (Fig. S4H) and tube formation (*P* = 0.01, Fig. S4I). Taken together, these results demonstrate that miR-146b-5p stimulates the pro-fibrotic transition of cardiac cells, possibly through *IRAK1* and *CEACAM1*.

### miR-146b-5p inhibition reduced cardiac fibrosis and apoptosis, promoted angiogenesis, and increased reparative macrophages in an MI mouse model

**Figure 4:**
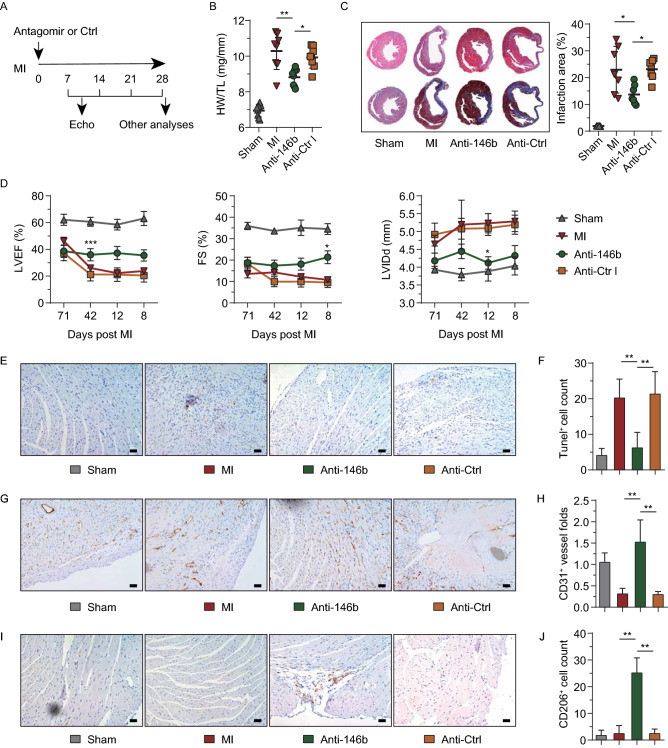
**Therapeutic modulation of miR-146b-5p restores cardiac function in the MI mouse.** (A) Workflow for animal experiments to determine the effect of miR-146b-5p inhibition on infarcted hearts *in vivo*. The miR-146b-5p antagomir (Anti-146b) or control (Anti-Ctrl) was locally administered after MI surgery. The cardiac structure and function were studied for up to 4 weeks. (B) The ratio of heart weight to tibia length (*n* = 8 per group). (C) Representative images for hematoxylin and eosin (HE) and Masson’s staining and quantification of infarcted area (*n* = 8 samples per group, 10 random fields per sample). (D) Echocardiographic analyses of cardiac functions at 7, 14, 21, and 28 days post-MI and antagomir treatment (*n* = 7 per group). (E and F) Representative images for TUNEL staining and quantification of TUNEL^+^ apoptotic cells. Blue signals indicate cell nuclei. Brown signals represent TUNEL^+^ apoptotic cells (*n* = 7 per group, 10 random fields per sample); bar = 50 μm. (G and H) Representative images for CD31 staining and quantification of CD31^+^ vessels. Blue signals indicate cell nuclei. Brown signals indicate CD31^+^ vessels (*n* = 7 per group, 10 random fields per sample); bar = 50 μm. (I and J) Representative images for CD206 staining and quantification of CD206^+^ reparative macrophages. Blue signals indicate cell nuclei. Brown signals indicate CD206^+^ macrophages (*n* = 7 per group, 10 random fields per sample); bar = 100 μm. Data are expressed as the mean ± SD. *P < 0.05 vs. Anti-Ctrl group; **P < 0.01. Data in panel (D) were analyzed using two-way ANOVA followed by Tukey’s post-hoc analysis. Other data were analyzed using one-way ANOVA followed by Tukey’s post-hoc analysis. HW, heart weight; TL, tibia length; LVEF, left ventricular ejection fraction; FS, fractional shortening; LVIDd, left ventricular internal diastolic diameter; Anti-146b, antagomir miR-146b-5p; Anti-Ctrl, antagomir control.

To assess whether miR-146b-5p is a viable therapeutic target for fibrotic remodeling following ischemic injury, an *in vivo* inhibition strategy was devised. Mice were treated with the miR-146b-5p antagomir immediately after MI surgery (Fig. 4A). Compared to the antagomir negative control group (Anti-Ctrl), the miR-146b-5p inhibition group (Anti-146b) exhibited a marked decrease in heart weight/tibia length (HW/TL, Fig. 4B), and the left ventricular (LV) wall thickness and infarction area were significantly restored (*n* = 8, *P* = 0.0001; Fig. 4C). Specifically, the Anti-Ctrl group had a higher HW/TL ratio (9.93% ± 0.56%) than the Anti-146b group (8.82% ± 0.45%). Moreover, the infarction area was 22.60% ± 3.56% and 13.12% ± 3.31% in the Anti-Ctrl and Anti-146b groups, respectively (*P* = 0.001). Echocardiography showed that miR-146b-5p inhibition restored the left ventricular ejection fraction (LVEF), fractional shortening (FS), and left ventricular internal diastolic diameter (LVIDd) (Fig. 4D). Significant differences in LVEF were observed at day 14 post-MI and remained stable to day 28 post-MI. The LVEF at 28 days post-MI were 20.43% ± 4.9% and 34.39% ± 3.8% in the Anti-Ctrl group and Anti-146b group, respectively (*P* = 0.01). Compared to the Anti-Ctrl group, the FS in the Anti-146b group was significantly higher at 28 days post-MI (*P* = 0.02). Despite only one significant difference being found regarding the LVIDd between these two groups at day 21, the average LVIDd in the Anti-Ctrl group remained higher than in the Anti-146b group (Fig. 4D).

TUNEL staining revealed that miR-146b-5p inhibition significantly reduced the number of apoptotic cells following MI from 21% ± 5.73% to 6% ± 3.94% (*P* = 0.0002; Fig. 4E and 4F). Immunohistochemical staining demonstrated that Anti-146b treatment significantly increased the number of CD31^+^ microvessels in the ischemic myocardium (Fig. 4G and 4H). In addition, we found CD206^+^ reparative macrophages to be increased by 9.38-fold following antagomir treatment compared to the Anti-Ctrl (Fig. 4I and 4J).

### Therapeutic silencing of miR-146b-5p improved cardiac fibrosis and ventricular strain in a porcine MI model

**Figure 5:**
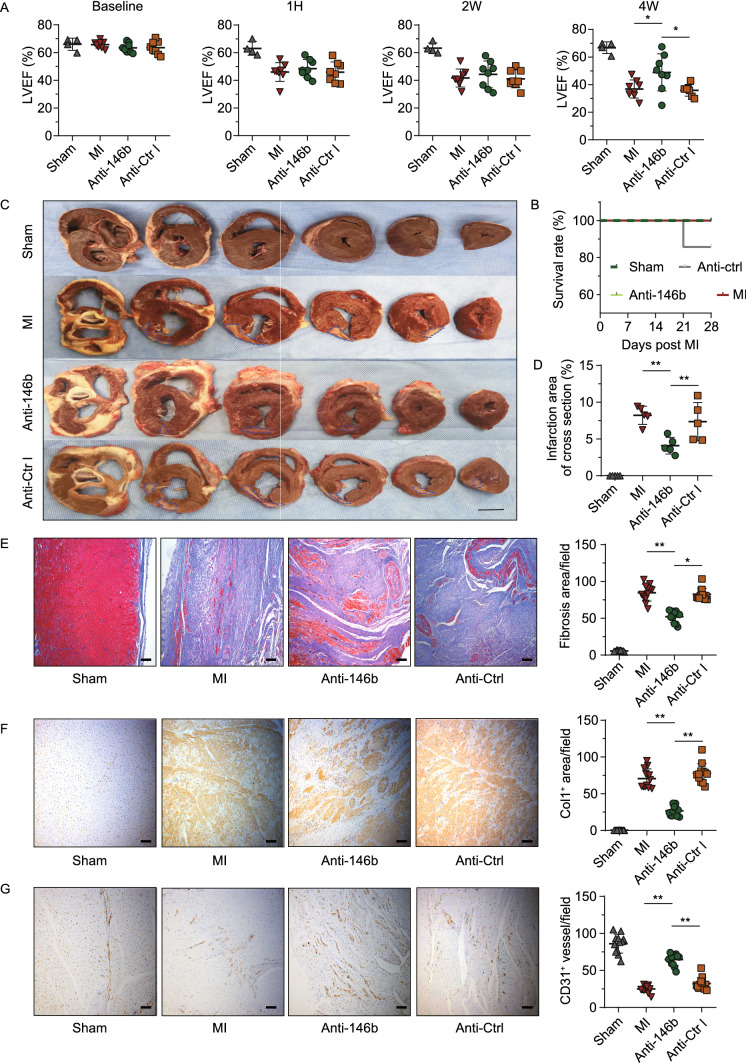
**Therapeutic modulation of miR-146b-5p relieves cardiac fibrosis and remodeling in MI pig.** (A) The LVEF of the experimental pigs was analyzed using echocardiography at different time points (sham group: *n* = 4, other groups: *n* = 8 each). (B) The survival analysis of the experimental groups. The survival curve was plotted using the Kaplan-Meier method. Pigs were sacrificed 4 weeks after MI. (C) Representative slices of the porcine myocardium. Infarction areas are indicated by the blue dashed line. Bar = 1 cm. (D) Infarction area analysis. Five whole heart sections per group were randomly selected, and the percentage of infarction area to total area in cross section was calculated. (E–G) The representative images for histological and immunohistochemical staining are shown (10 random fields per group, bar = 100 μm), indicating fibrosis, as per Masson’s staining (E); expression of collagen type 1 (Col1, F); and CD31^+^ capillaries (G). Cell apoptosis was analyzed by TUNEL staining (Fig. S5). Data are expressed as the mean ± SD. *P < 0.05; **P < 0.01. Data were analyzed using one-way ANOVA followed by Tukey’s post-hoc analysis. 1H, 1 h; 2W, 2 weeks; 4W, 4 weeks; Anti-146b, antagomir miR-146b-5p; Anti-Ctrl, antagomir control

**Figure 6:**
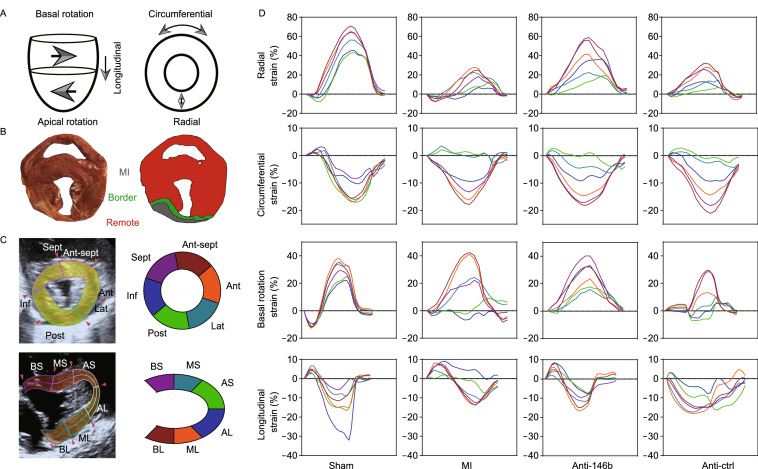

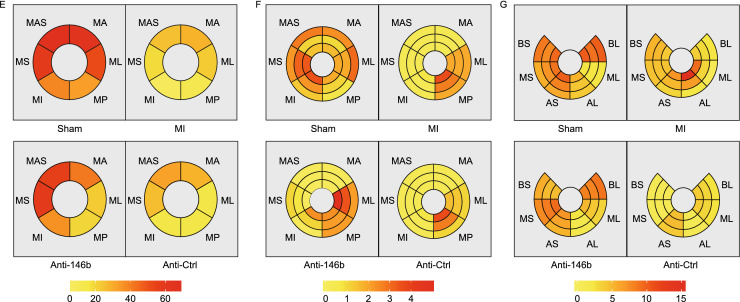
**Therapeutic modulation of miR-146b-5p improves myocardial strains in MI pig.** (A) The cardiac strains in different directions. (B and C) The definition of myocardial segmentation and the representative echocardiographic images of pig heart obtained 4 weeks post-MI (at end systole). (C) For speckle tracking, the left ventricle is divided into six anatomical zones in both the cross-sectional and longitudinal direction. (D) Representative segmental strain curves of the left ventricular (LV) mid-wall over one cardiac cycle at 4 weeks post-MI in the longitudinal, radial, circumferential, and rotation directions. (E–G) Bull’s eye diagrams of the absolute value of peak myocardial segmental strain in the radial (E), circumferential (F), and longitudinal (G) directions at 4 weeks post MI. BS, basal septum; MS, mid septum; AS, apical septal; AL, apical lateral; ML, mid lateral; BL, basal lateral; Ant-sept, anterior septum; Ant, anterior; Lat, lateral; Post, posterior; Inf, inferior; Sept, septum; Anti-146b, antagomir miR-146b-5p; Anti-Ctrl, antagomir control

We next studied the therapeutic potential of miR-146b-5p in a porcine model. A small-scale MI was induced by left circumflex artery (LCX) ligation surgery in minipigs, as developed and validated by Shen et al. (1999). A homologous antagomir was used for the porcine study. After ligation, 200 nmol antagomir in 1 mL PBS was immediately injected into the ischemic myocardium and adjacent border zone. The porcine cardiac structure and functions were evaluated by histological assays and echocardiography (Figs. 5, 6; Tables 1 and 2). Echocardiography indicated a marked improvement of cardiac function with miR-146b-5p silencing four weeks after MI. The LVEF in the antagomir-treated pigs (Anti-146b) was restored to 48.89 ± 13.45, while the LVEF in the antagomir control pigs (Anti-Ctrl) decreased to 37.26 ± 5.47 (*n* = 8, *P* = 0.039; Fig. 5A). Detailed echocardiographic results were showed in Table 1. Only one experimental animal in the antagomir control group died during the study (at day 21) (Fig. 5B). Morphologic observation and Masson staining of cardiac sections showed that antagomir treatment (Anti-146b) significantly reduced the infracted area from 7.13% ± 2.33% to 3.59% ± 1.02% (% of entire cross-section area; *P* = 0.02 vs. Anti-Ctrl) (Fig. 5C–E). Immunohistochemical staining demonstrated that miR-146b-5p antagomir treatment significantly decreased the expression of collagen type 1 and increased the number of CD31^+^ microvessels in the porcine MI heart, from 70.50 ± 12.80 to 26.86 ± 6.35 (*P* = 0.0001) and 24.92 ± 4.59 to 64.67 ± 7.74 (*P* = 0.0001), respectively, when compared with the Anti-Ctrl group (Fig. 5F and 5G). TUNEL staining demonstrated that miR-146b-5p inhibition markedly reduced the number of TUNEL^+^ apoptotic cells following MI from 70.57 ± 16.43 to 44.71 ± 19.39 TUNEL^+^ cells per view (*P* = 0.02; Fig. S5).

**Table 1:** Echocardiographic data of experimental pigs

	Baseline				4wks			
	Sham	MI	Anti-146b	Anti-Ctrl	Sham	MI	Anti-146b	Anti-Ctrl
EF	65.98 ± 4.39	65.73 ± 2.54	63.38 ± 3.46	63.56 ± 4.74	66.85 ± 4.24	36.85 ± 6.56^‡^	48.89 ± 13.45^‡,#,§^	37.26 ± 5.47^‡^
LVIDd	30.78 ± 1.89	34.46 ± 4.68	35.86 ± 3.42	31.90 ± 5.63	33.95 ± 4.53	45.90 ± 4.49	39.36 ± 5.13	41.33 ± 1.10^‡^
LVIDs	19.95 ± 1.84	22.31 ± 3.15	23.80 ± 2.58	21.06 ± 2.88	21.73 ± 3.43	31.33 ± 3.54^†^	30.89 ± 4.90^†^	33.20 ± 4.08
LVPWd	6.15 ± 0.45	6.25 ± 0.61	6.55 ± 1.14	6.75 ± 0.93	7.60 ± 1.47	4.50 ± 0.59^‡^	6.18 ± 0.71^#,§^	4.69 ± 1.16^‡^
LVPWs	8.58 ± 0.95	9.53 ± 0.83	9.41 ± 0.49	9.38 ± 1.97	10.95 ± 1.14	5.62 ± 1.15^‡^	9.41 ± 0.49^**#,||**^	5.59 ± 1.28^‡^
LVEDV	47.40 ± 6.33	50.24 ± 15.75	51.10 ± 9.30	52.03 ± 12.33	53.28 ± 9.63	64.90 ± 2.86	56.63 ± 6.14	61.88 ± 2.86^†^
LVESV	12.80 ± 3.06	17.33 ± 5.77	18.48 ± 3.93	17.44 ± 3.67	16.25 ± 6.12	44.23 ± 5.42^‡^	30.74 ± 8.52^‡,**#,**§^	39.70 ± 1.55^‡^

Data was shown as mean ± SD

“**†**” *P* < 0.03 vs. sham; “**‡**” *P* < 0.01 vs. sham; “**#**” *P* < 0.05 vs. MI; “**§**” *P* < 0.03 vs. Anti-Ctrl; “**||**” *P* < 0.01 vs. Anti-Ctrl

**Table 2:** Peak strain (%) of pigs 4 weeks after MI induction

		Sham	MI	Anti-146b	Anti-Ctrl
Longitudinal strain	BL	33.26 ± 4.31	9.42 ± 1.52^‡^	7.52 ± 1.54^‡^	7.44 ± 0.70^‡^
	ML	15.62 ± 2.10	7.38 ± 0.87^‡^	7.74 ± 1.15^‡^	7.28 ± 0.89^‡^
Radial stain	Inf	48.58 ± 6.76	8.90 ± 3.43^‡^	36.04 ± 2.52^‡,*,§^	13.70 ± 3.05^‡^
	Post	44.88 ± 5.48	12.38 ± 0.94^‡^	20.70 ± 1.36^‡,*,§^	7.58 ± 1.16^‡^
Circumferential strain	Inf	10.66 ± 1.36	9.54 ± 0.83	9.58 ± 1.10	9.60 ± 0.63
	Post	17.16 ± 1.60	2.72 ± 0.81^‡^	5.58 ± 1.18^‡,*,§^	3.04 ± 0.49^‡^
Rotation strain	Inf	24.40 ± 2.31	6.30 ± 2.45^‡^	24.32 ± 0.97^*,§^	5.44 ± 1.59^‡^
	Post	24.40 ± 0.47	10.04 ± 1.23^‡^	16.38 ± 4.54^‡,*,§^	6.56 ± 1.76^‡^

Data was shown as mean ± SD

BL, basal lateral; ML, mid lateral; Inf, inferior; Post, posterior

“**†**” *P* < 0.03 vs. sham; “**‡**” *P* < 0.01 vs. sham; “*” *P* < 0.03 vs. MI; “**#**” *P* < 0.01 vs. MI; “**§**” *P* < 0.03 vs. Anti-Ctrl; “**||**” *P* < 0.01 vs. Anti-Ctrl

Speckle tracking echocardiography (STE) analysis is a novel way to evaluate ventricular wall motion in large animals (Torres et al., 2018). Our study indicated that miR-146b-5p inhibition significantly improved the cardiac strain and ventricular wall motion of the ischemic myocardium four weeks after MI (Fig. 6). Figure 6A–C shows the cardiac strain classification, infarcted area, and segment definition of experimental hearts. In Fig. 6D, each line represents the strain changes of relevant segments during all phases of the cardiac cycle. We found that radial, rotation, and part circumferential strains were significantly improved in the Anti-146b group compared to the MI and Anti-Ctrl groups. No significant improvement was observed regarding the longitudinal strain among the groups. Figure 6E–G shows a bulls’ eye diagram with each segment in the cross section of the myocardium labelled according to its peak strain (%) at four weeks post-MI, in three directions: radial (E), circumferential (F), and longitudinal (G). Details regarding peak myocardial strains (%) of the ischemic area are shown in Table 2. Taken together, these data demonstrate that silencing miR-146b-5p significantly alleviates cardiac fibrosis and cell death to enhance angiogenesis and cardiac function in a porcine MI model.

## Discussion

In adult mammals, the resident heart cells have been demonstrated to be key cellular effectors in the inflammatory response during cardiac injury and repair (Westman et al., 2016; Fu et al., 2018). In the present study, we found that miR-146b-5p expression was upregulated in fibroblasts, endothelial cells, and macrophages under hypoxic conditions. Cardiac fibrosis is predominantly orchestrated by myofibroblasts derived from FMT (Prabhu and Frangogiannis, 2016). The TGFβ-Smad signaling pathway is the core signaling pathway governing FMT. Nagpal et al. demonstrated that inhibition of FMT by targeting TGF-β-induced miR-125b expression successfully prevented fibrotic remodeling after MI (Nagpal et al., 2016). In the present study, miR-146b-5p was found to be a novel facilitator of FMT; it was activated by hypoxia rather than by the classic pro-fibrotic TGF-β signaling.

CMVECs play a vital role in the regulation of fibrotic remodeling (Liu et al., 2019). Previous work found that miR-146 represses endothelial cell activity by targeting endothelial nitric oxide synthase in response to stimulation by the pro-inflammatory cytokine IL-1β (Cheng et al., 2013). We revealed that miR-146b-5p impaired CMVEC tube formation in Matrigel and reduced cell viability under starvation *in vitro*. By using an antagomir, we found that ischemic myocardium with inhibited miR-146b-5p expression showed a marked increase in the number of CD31^+^ microvessels (about 6-fold compared to control). Due to the local upregulation of IL-1β following MI, miR-146b-5p inhibition may preserve cardiac endothelial cell activity and function, and our data provides direct evidence for this *in vivo*.

Cardiac macrophages participate in homeostatic maintenance and tissue repair by producing reparative inflammatory mediators or undergoing polarization (Shiraishi et al., 2016; Ferraro et al., 2019). Some pioneering studies have reported that miR-146b regulates macrophage paracrine signaling and activation under various inflammatory environments (He et al., 2016; Peng et al., 2016; Deng et al., 2019; Zhang et al., 2019); however, little is known about the effects of miR-146b on cardiac macrophages. In the present study, the four most abundant cytokines in wounds (IL-1β, IL-6, TNF-α, and MCP-1) (Mescher, 2017) were found to be significantly reduced in the presence of a miR-146b-5p mimic, whereas many were significantly increased by treatment with an miR-146b-5p inhibitor. Figure 4I and 4J show the first direct *in vivo* evidence of miR-146b-5p inhibition increasing the number of reparative CD206^+^ macrophages, which may also aid in the repair of the ischemic myocardium.

The human miR-146 family is composed of two genes: *MIR146A* and *MIR146B*. These two miRNAs are located on different chromosomes and have differential regulatory functions (Paterson and Kriegel, 2017). Most published literature has focused on the effect of the miR-146 family in cancer or the immune system, and there are only a few reports of its functions in cardiovascular disease (Gao et al., 2015; Wang et al., 2015; Cheng et al., 2017; Desjarlais et al., 2019). Specifically, Di et al. found that miR-146b was upregulated in the ischemia/reperfusion(I/R) myocardium. miR-146b overexpression reduced the infarct size in the I/R model and attenuated I/R-induced H9C2 apoptosis *in vitro* (Di et al., 2017). Li et al. found that miR-146b protected H9C2 cells against hypoxia (Li et al., 2015). These contrasting results may be explained by our use of primary neonatal cardiomyocytes rather than a cell line, and our use of a permanent MI model rather than an I/R model. Furthermore, our data demonstrated that miR-146b-5p expression was not significantly changed in primary cardiomyocytes after 24 h under hypoxic conditions, suggesting that the upregulation of miR-146b detected in the I/R myocardium may be derived from non-cardiomyocyte cells, such as fibroblasts. Additionally, the mature miR-146b includes miR-146b-3p and -5p; each strand has a unique binding site and function. We investigated miR-146b-5p in the present study. These differences in experimental settings may have led to different results. Here, we found that miR-146b-5p regulates the reparative phenotype of cardiac cells and demonstrated the feasibility of alleviating cardiac remodeling and dysfunction by inhibiting miR-146b-5p in both small (mice) and large (pig) mammals. To the best of our knowledge, this is the first study investigating the therapeutic potential of miR-146b-5p on cardiac remodeling and dysfunction in a pig MI model.

In recent years, pioneering studies have demonstrated that the silencing of specific microRNAs, including miR-199a (Gabisonia et al., 2019), miR-15 (Hullinger et al., 2012), miR-21 (Thum et al., 2008), miR-92a (Hinkel et al., 2013), and miR-125b (Nagpal et al., 2016) reduces cardiac fibrosis and dysfunction *in vivo*. In the present study, we found that miR-146b-5p expression was regionally increased in the ischemic myocardium; we then injected a miR-146b-5p antagomir into the infarcted and peri-infarcted myocardium. Our *in vivo* study of antagomir efficacy identified a single dosage that significantly inactivated the effects of miR-146 on target genes for up to three days (Fig. S6), suggesting a short-term inhibition of miR-146b-5p was achieved in the present study. Given that miR-146b is an anti-inflammatory factor, its long-term inhibition may prevent the timely resolution of inflammation following MI, which has been demonstrated to be detrimental. In other words, the dosage and intervention time must account for the biphasic nature of cardiac inflammation following MI. In murine models, reparative inflammatory signals were triggered within the first four days after MI. We thus postulate that the effective window for miR-146b-5p intervention should not exceed the inflammatory phase immediately after MI.

Although promising, there are remaining hurdles that must be overcome to achieve this goal in clinical settings. One major concern is the pleiotropic nature of microRNAs (Mendell and Olson, 2012). Although local treatment (Hinkel et al., 2013; Yang et al., 2018a, b) can partially prevent the risk of undesired side effects induced by systemic anti-microRNA treatment, current anti-microRNA therapy is not yet cell-specific and results in a general systemic inhibition of microRNA expression. Although the above-mentioned technical hurdles have not yet been addressed, this study provides a basis for improving our current understanding of immunomodulatory microRNAs and fibrotic remodeling post-MI in large mammals. Our data indicate that targeting immunoregulatory microRNAs may represent a novel therapeutic strategy for complementary use with current ischemic heart disease medications.

## Materials and methods

### Human samples

Human blood samples were collected from Shanghai East Hospital and Zhongshan Hospital with informed consent and were approved by the hospital ethics committee in accordance with the Helsinki Declaration. Arterial blood (5 mL) was drawn from patients diagnosed with CTO through a catheter before percutaneous recanalization. Equivalent blood samples were obtained from health volunteers by arteriopuncture. Clinical parameters and medical histories were available for all patients (Table S1).

### Animal studies

All experimental procedures were approved by the local Institutional Animal Care and Use Committee and performed in accordance with the related guidelines.

### Myocardial infarction surgery and antagomir treatments

Male C57BL/6 mice aged 10–12 weeks were purchased from Shanghai SLAC Laboratories. Surgery was carried out as previously described (Hinkel et al., 2013; Yang et al., 2018a, b). Briefly, mice were anesthetized with isoflurane (2%) and ventilated using a rodent ventilator (Harvard Apparatus Inc.) throughout the surgical procedure. The descending coronary artery was ligated with a silk suture (Nakada et al., 2017). The chest was then closed in layers. Sham-operated animals underwent the same procedure without ligation. For *in vivo* miRNA treatment, a total of 20 nmol miR-146b-5p antagomir (miR30003475-4-5, Ribobio, China) or negative control antagomir (miR3N0000001-4-20, Ribobio, China) was dissolved in 40 µL of PBS and injected into the infarct and peri-infarct zone using a syringe (Nakada et al., 2017). Echocardiography was performed before and after left anterior descending coronary artery ligation using a Supersonic Aixplorer Vevo 2100 system.

Female Bama minipigs (6 months, 20 kg) were randomly assigned to the following groups: sham group (Sham, *n* = 4); MI + saline group (MI, *n* = 8); MI + miR-146b-5p antagomir group (Anti-146b, *n* = 8); and MI + antagomir negative control group (Anti-Ctrl, *n* = 8). The surgical procedure to induce myocardial ischemia has been described previously (Shen et al., 1999; Purcell et al., 2014). Briefly, the animals were anesthetized using 2% isoflurane, intubated, and ventilated using an animal anesthesia ventilator (Matrx model 3000; USA). The left circumflex artery (LCX) was ligated with a 4-0 silk suture. Commercially available miR-146b-5p antagomir or negative control antagomir (200 nmol in 1 mL PBS; miR30003475-4-5 and miR3N0000001-4-20, Ribobio, China) was injected intramyocardially into the infarct and peri-infarct zone using a syringe (Krutzfeldt et al., 2005). The chest was then closed in layers and the animals were allowed to recover. Standard post-operative care was administered until the animals fed normally. One hour after surgery, an echocardiograph was performed to assess cardiac function. Postoperative animals with LVEF ranging from 30% to 60% were enrolled and followed up for four weeks. Three animals died of lethal arrhythmia during surgery.

### Cardiac function analyses

Mouse cardiac function was assessed according to a previously published protocol (Yuan et al., 2018). For minipigs, cardiac function was measured using a TOSHIBA Artida/SSH-880CV. Briefly, minipigs were anesthetized and placed in the supine position. The end-diastolic and end-systolic volumes (LVEDV and LVESV) and LVEF were calculated using the Simpson method. Wall thickness (LVPWd and LVPWs) and left ventricle diameter (LVIDd and LVIDs) were measured at early and end-diastole in the oblique right parasternal long-axis, four-chamber view. STE analysis was performed as previously described (Torres et al., 2018). Continuous strain measurements of different ventricle segments over the whole cardiac cycle were obtained.

### Histology and immunostaining

Heart samples were harvested, fixed with paraformaldehyde, and embedded in paraffin. Hematoxylin and eosin (HE) and Masson’s trichrome staining were performed. To quantify the fibrosis area, ten fields of view were randomly selected from eight cardiac sections and the percentage of Masson’s trichrome positive-stained area relative to total myocardial area was calculated. TUNEL staining was performed using a TUNEL apoptosis detection kit (40306ES50, Yeasen, China), according to the manufacturer’s instructions. Immunohistochemical staining was performed according to the standard protocol and using the following antibodies: Collagen type 1 (Col I, 1:1,000; ab34710, Abcam), αSMA (1:500; #19245, CST), CD31 (1:1,000; ab182981, Abcam), CD206 (1 µg/mL; ab64693, Abcam), goat anti-rabbit IgG H&L (HRP) (1:10,000; ab205718, Abcam), and goat anti-mouse IgG H&L (HRP) (1:10,000; ab205719, Abcam). For immunofluorescence, cells were fixed in cold methanol for 5 min, then permeabilized and blocked in phosphate buffered solution (0.5% Triton X-100) with 1% bovine serum albumin for 1 h. Immunostaining was performed using the following antibodies: Collagen type 1 (ab34710, Abcam), αSMA (#19245, CST), and the corresponding secondary antibodies.

### Cell culture and treatments

The primary culture of mouse neonatal cardiomyocytes and cardiac fibroblasts was performed as we have previously published (Liu et al., 2019). In brief, neonatal cardiomyocytes were isolated from 1-day-old C57BL/6 mice by enzyme digestion (0.06% trypsin and 0.025% collagenase). Single cell suspensions were plated on 100-mm culture dishes in high-glucose Dulbecco’s modified Eagle’s medium (DMEM) with 14% fetal bovine serum (FBS) for 2 h. Non-attached cardiomyocytes were transferred to another dish to culture and 100 μmol/L bromodeoxyuridine was added to block fibroblast proliferation. The attached cardiac fibroblast fraction was cultured in DMEM with 10% FBS and 1% penicillin/streptomycin. Cells were cultured in serum-free medium for 4 h before treatment. NIH3T3 fibroblasts were obtained from the ATCC and cultured following the classic protocol (Peres et al., 2018).

CMVECs were isolated as previously described (Liu et al., 2019). Briefly, minced mouse myocardium tissue was digested using 1 mg/mL warm collagenase type 1 (C5849, Sigma), 5 units/mL dispase (345235, Collaborative), and 60 units/mL DNase 1 (10104159001, Roche) for 45 min. After filtration through a 70-μm cell strainer, single cell pellets were incubated with anti-CD31 magnetic microbeads (antibody: 553370, BD; microbeads: 11035, Invitrogen) for 20 min following the manufacturer’s instructions. After magnetic separation, CMVECs were cultured in endothelial cell growth medium (CC-3162, Lonza).

Mouse cardiac macrophages were isolated as previously described (Jia et al., 2019), with modifications. Specifically, two days after MI surgery, the myocardium of the left ventricular anterior wall was minced and enzymatically digested with 1.5 mg/mL collagenase type 2, 0.25 mg/mL elastase, and 0.5 mg/mL DNase 1 for 1 h at 37 °C. The cell suspension was passed through a 70-μm cell strainer and macrophages were isolated by positive selection using CD11b (130-049-601, Miltenyi Biotec) and F4/80 microbeads (130-110-443, Miltenyi Biotec) with a magnetic cell sorting separator (Miltenyi Biotec) according to the manufacturer’s protocol.

A commercially available miR-146b-5p mimic (miR10003475-1-5, Ribobio) and inhibitor (miR20003475-1-5, Ribobio) were used for gain- and loss-of-function experiments *in vitro* according to the manufacturer’s protocol. Dose-response curves were determined using cardiac fibroblasts at 10, 20, and 50 nmol. *In vitro* hypoxia experiments were conducted using Genbag (45534, BioMerieux). Cells were incubated under hypoxic conditions for 24 h.

### Cell proliferation analyses

Cell proliferation analyses were performed using cell counting kit-8 (CCK-8) (CK04-11, Dojindo, Japan) and the Cell-Light EdU kit (C10310-3, Ribobio, China). Briefly, 100 μL of cell suspension containing 3,000 cells was added to a 96-well plate. Fibroblasts were transfected with miR-146b-5p mimic, miR-146b-5p inhibitor, or the control (Ctrl) using the riboFECT^™^ CP Transfection Kit (C10511-1, Ribobio) according to the manufacturer’s instructions and cultured for an additional 24 h. For CCK-8 analysis, 10 μL of the CCK-8 reagent was added to the medium and incubated for 3 h. The OD levels were measured using a spectrophotometric microplate reader at 450 nm. For EdU analysis, the reagent was added to the medium and incubated for 2 h to label EdU. Cell images were then acquired at five random fields per sample using a Leica fluorescence microscope. The data presented are the average of three independent assays performed in triplicate.

### Cell migration analyses

Cell migration analyses were performed via a Transwell assay (8.0 μm; 3422, Corning, USA) and a scratch wound assay. Fibroblasts were transfected with the miR-146b-5p mimic, miR-146b-5p inhibitor, or control using the riboFECT^™^ CP Transfection Kit (C10511-1, Ribobio) according to the manufacturer’s instructions. Transfected cells were harvested and resuspended in serum-free DMEM. An aliquot (200,000 cells/200 µL) of cells in serum-free DMEM was dispensed into the Transwell inserts, and DMEM with 20% serum was placed in the lower chamber. After 8 h of culture, the non-migrated cells were carefully removed and the migrated cells in the bottom were stained with crystal violet solution. Images were captured at five random fields by microscopy. The migrated cells were counted by two volunteers blinded to the study.

For the scratch wound assay, fibroblasts were seeded into 6-well plates and transfected with the miR-146b-5p mimic, miR-146b-5p inhibitor, or control as described above. The transfected cells were subjected to serum starvation for 24 h after reaching confluence. The cells were scratched in a crisscross manner and rinsed with DMEM to remove any cellular debris prior to culturing in DMEM supplemented with 10% FBS. The wound area was documented at 0 h (A) and 6 h (B) by photomicrographic images and measured with ImageJ software by two experienced investigators who were blinded to the treatments. The wound width percentages were calculated as: (A−B)/A ×100%.

### *In vitro* endothelial function assays: starvation, survival, and tube formation

*In vitro* endothelial function assays were performed as previously described (Kilic et al., 2005; Li et al., 2018). Briefly, CMVECs were transfected with the miR-146b-5p mimic, miR-146b-5p inhibitor, or control. Cells were cultured in normal endothelial growth medium until reaching confluence. The full medium was then replaced with starvation medium (DMEM containing 2% FBS without other supplements). The starvation medium was renewed every 3 days. After 3 days of culture, cell viability was assessed morphologically using phase-contrast microscopy. The criteria for viability were the state of confluence and the adherence of endothelial cells.

In the tube formation assay, 2 × 10^4^ mouse CMVECs were seeded onto 96-well plates pre-coated with Matrigel and incubated for 6 h. Tube formation was quantified by counting the number of loops and branch points, then calculating the total tube length. Five random fields were captured per group and three independent experiments were performed and analyzed.

### Cytokine measurement

Cardiac macrophages were cultured in the absence of cytokines and were transfected with the miR-146b-5p mimic, miR-146b-5p inhibitor, or control, as described previously (Yang et al., 2018a, b). The culture supernatant was collected two times—immediately after transfection was completed, and 6 hours’ culture following transfection completion. The levels of interleukin-1β (IL-1β), IL-6, tumor necrosis factor-α (TNF-α), and monocyte chemotactic protein 1(MCP-1) in the culture supernatant were measured using commercially available ELISA kits, according to the manufacturer’s instructions. All samples were assayed in triplicate.

### qPCR

qPCR was performed as previously described (Yang et al., 2018a, b), with minor modifications. In brief, total RNA was collected using Trizol reagent (Invitrogen). Long RNA was reverse transcribed using the PrimeScript^TM^ RT reagent kit with gDNA Eraser (RR047A, TaKaRa). For miRNA, a miRNA cDNA synthesis kit (A11193-051, Invitrogen) was used for polyadenylation and reverse transcription. qPCR was performed using TB Green^®^ Advantage^®^ qPCR Premix (639676, Clontech) on a QuantStudio^TM^ 6 Flex Real-time PCR System according to the manufacturer’s protocol. The miRNA primers were purchased from Ribobio (Guangzhou, China), hsa-miR-146b-5p primers (MQPS0000653-1-200), mmu-miR-146b-5p primers (MQPS0002464-1-200), and U6 primers (MQPS0000002-1-200) were adopted. Additional primers used for qPCR are listed in Table S2. The relative mRNA or miRNA expression levels were normalized to GAPDH or U6 snRNA, respectively, using the delta-delta Ct method. Three biological replicates per group were used for qPCR.

### Western blot

Cells were lysed in RIPA lysis buffer supplemented with protease inhibitor pellets (Amersham Biosciences, USA). Insoluble debris were removed by centrifugation (25,000 ×*g*) for 30 min at 4 °C. Cell lysates were boiled with loading buffer for 10 min. Equal amounts of protein (20 μg) were separated by SDS-PAGE and assessed by Western blot for the indicated protein(s) using the antibodies described above. Experiments were repeated three times and GAPDH was used as the loading control.

### Luciferase reporter construct and assay

The luciferase reporter assay was performed using the Dual-Luciferase® reporter assay kit (Promega, USA) and Promega GloMax 96 according to the manufacturer’s protocol. The pGM-NF-kappa B-Luc plasmid was purchased from Genomeditech (GM-021001, China). To assemble the 3′UTR reporter, the binding sites of miR-146b-5p on mouse *IRAK1* and *CEACAM1* mRNA 3′UTRs were amplified and cloned into a pGL3-promoter vector (Promega) to construct the wild-type (wt) and mutant (mut) luciferase reporters. Fibroblasts were co-transfected with individual reporter vectors (Luc-*CEACAM1*-wt, Luc-*CEACAM1*-mut, Luc-*IRAK1*-wt, Luc-*IRAK1*-mut; 0.2 μg/2.5 × 10^4^ cells) and control or miR-146b-5p mimics (20 nmol/L) using the riboFECT^™^ CP Reagent (C10511-1, Ribobio), according to the manufacturer’s instructions. Luciferase and *Renilla* activities were measured 48 h after transfection using a standard protocol. The relative luciferase unit was defined as the ratio of luciferase versus *Renilla* activity compared to that of the control (set to 1).

### Statistical analysis

Data are presented as the mean ± standard deviation (SD) for at least three independent assays unless otherwise noted. Student’s *t*-test was used to compare the differences between two groups. One-way ANOVA with Tukey’s post-hoc test was used for comparisons between multiple groups. The survival curve was plotted using the Kaplan-Meier method. GraphPad Prism 8 was used for the statistical analyses. Differences with *P*-values < 0.05 were considered statistically significant.

## Author contributions

Concepts, H.Z. and J.G.; resources, H.Z. and Z.L; experiments and data analysis, Y.L., H.C., H.L., and Y.D.; manuscript preparation, Y.L., H.L. and H.Z.; revision and editing, all authors.

## Electronic supplementary material

Below is the link to the electronic supplementary material.

Supplementary material 1 (PDF 3486 kb)
