# Supplementary material for: Therapeutic silencing miR-146b-5p improves cardiac remodeling in a porcine model of myocardial infarction by modulating the wound reparative phenotype
Source: Protein Cell. 2020 Aug 26;12(3):194–212. doi: 10.1007/s13238-020-00750-6 (PMC7895884; doi:10.1007/s13238-020-00750-6)
Supplement: Supplementary file 1 — Supplementary material 1 (PDF 3486 kb) [file 13238_2020_750_MOESM1_ESM.pdf]

Supporting Data

Therapeutic Silencing miR-146b-5p Improves Cardiac Remodeling in a Porcine Model of Myocardial Infarction by Modulating the Wound Reparative Phenotype

Supplementary Figure:

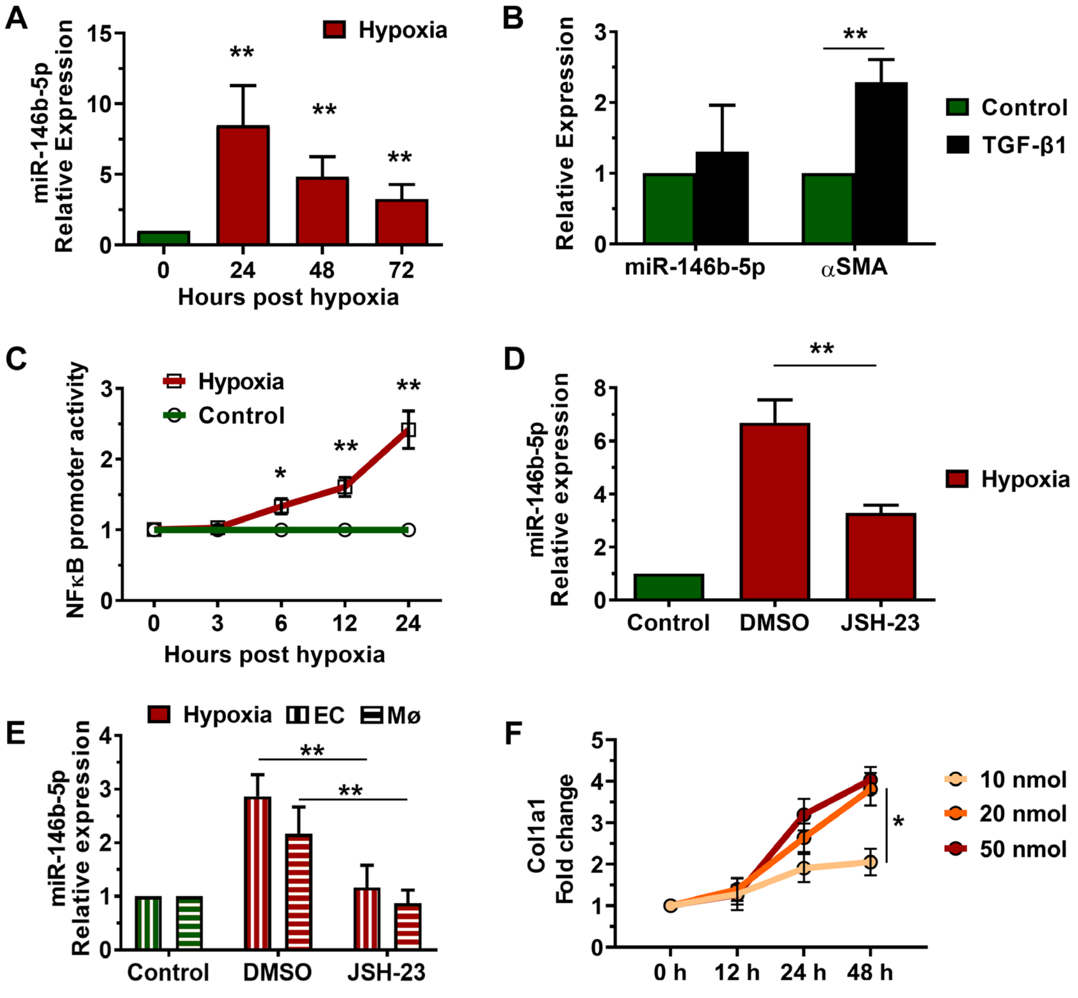

Figure S1. The miR-146b-5p was induced by hypoxia, instead of TGF-β, in a NF-kappa-B-dependent manner.

(A) The expression level of miR-146b-5p in primary cultured fibroblasts after hypoxia treatment were measured by qPCR (n=5). **(B)** After 24 h of treatment with 10 ng/ml TGF-β, the level of miR-146b-5p and *αSMA (ACTA2)* were measured by qPCR (n=5). **(C)** The NF-κB promoter activity were measured by the luciferase reporter assay (n=5). The commercially available pGM-NF-kappa B-Luc plasmid was purchased and

transfected into fibroblasts. The relative luciferase unit (RLU) was defined as the ratio of luciferase versus *Renilla* activity in the sample compared to that of the control. **(D)** The level of miR-146b-5p in fibroblast after hypoxia and NF-κB transcriptional inhibitor (JSH-23, 30 μM) treatment were measured by qRT-PCR after 24 h of treatment (n=5). **(E)** The levels of miR-146b-5p in endothelial cells and macrophages after hypoxia and JSH-23 (30 μM) treatment were measured by qRT-PCR after 24 h of treatment (EC, endothelial cell; Mø, macrophage; n=5). **(F)** In vitro dose-response study of the miR-146b-5p mimic, based on *COL1A1* expression in fibroblasts (n=5). The NIH3T3 fibroblasts were used in B-D. Primary cultured mouse fibroblast were used in A and F. Data are expressed as the mean ± SD. Data in A, B and C were analyzed using Student's t-test (*, $P<0.05$ vs control; **, $P<0.01$ vs control). Data in D and E was analyzed using one-way ANOVA followed by Tukey's post-hoc analysis (*, $P<0.05$; **, $P<0.01$). Data in F was analyzed by two-way ANOVA followed by Bonferroni post-hoc analysis (*, $P<0.05$).

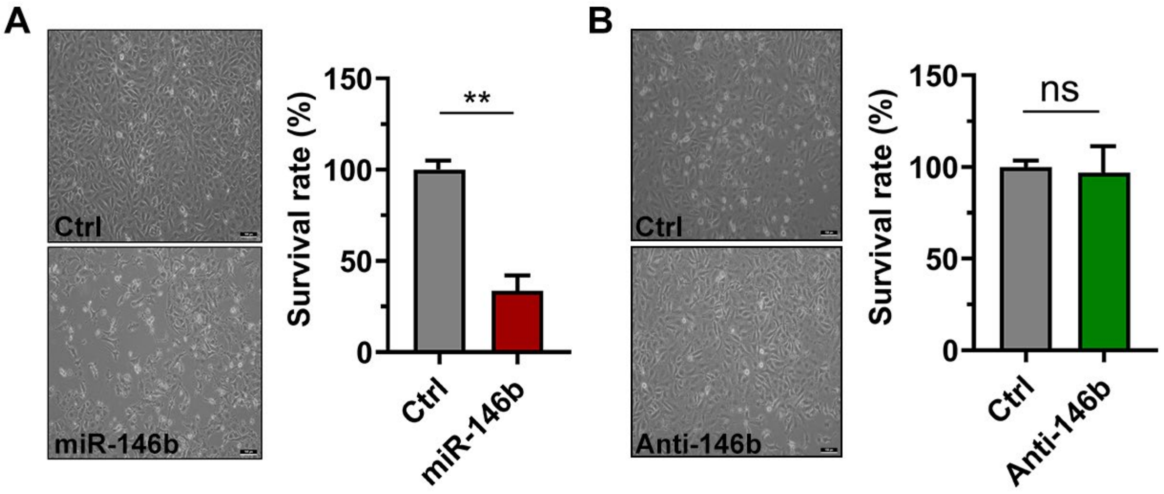

Figure S2. Upregulation of miR-146b-5p deteriorated survival of cardiac microvascular endothelial cells (CMVECs) under starvation conditions.

CMVEC cells in starvation medium were treated with miR-146b-5p mimic (miR-146b, A), miR-146b-5p inhibitor (Anti-146b, B), or control (Ctrl). After 3 days of starvation culture, the percentage of adhered endothelial cells was counted (n=7). **, P<0.01 vs control; ns, not significant. Data were analyzed using Student's t-test; they are expressed as the mean ± SD. Bar=100 μm.

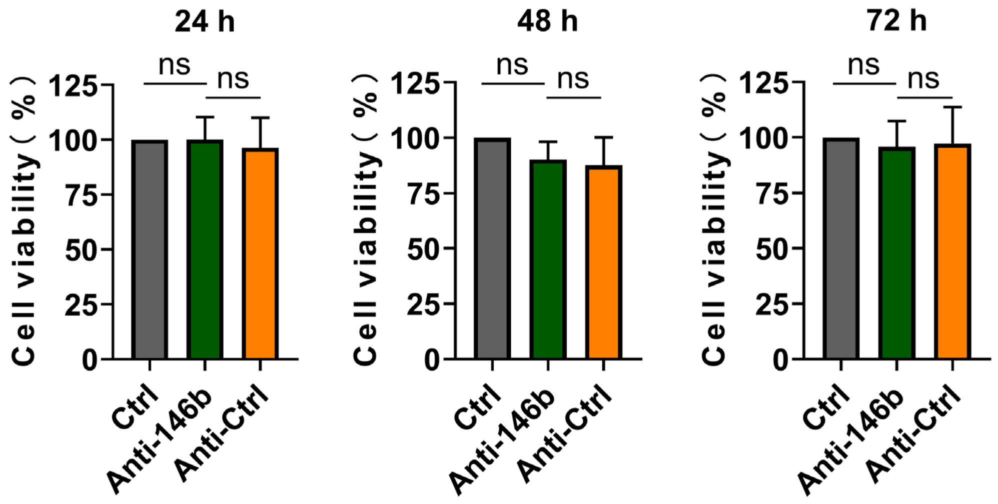

Figure S3. Inhibition of miR-146b-5p has no significant effect on cardiomyocyte viability in vitro.

After treatment with 20 nmol of miR-146b-5p inhibitor (Anti-146b) or control (Aniti-Ctrl), the cell viability of primary cultured neonatal cardiomyocytes was measured by CCK-8 at different time points (n=8). Data are expressed as the mean ± SD. ns, not significant. Data were analyzed using Student's t-test.

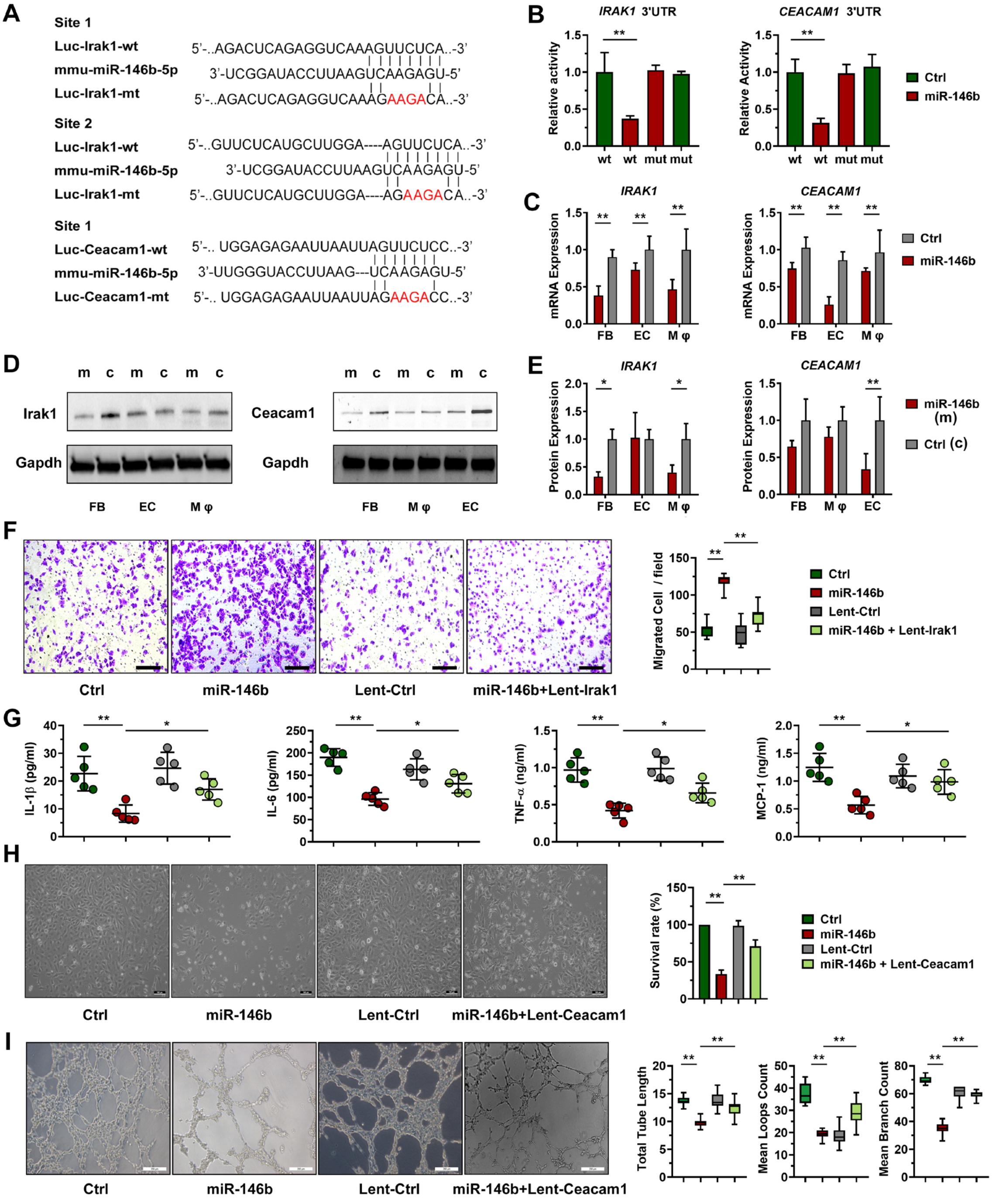

Figure S4. miR-146b-5p may modulate cardiac cell phenotypes by targeting *IRAK1* and *CEACAM1*

(A) The predicted binding sites of miR-146b. The mutation sites in sequences are in red. **(B)** The luciferase assays were performed in NIH3T3 fibroblasts co-transfected with wild-type or mutated 3'UTR of *IRAK1* and *CEACAM1* (n=5 per group). **(C-E)** Expression levels of *IRAK1* and *CEACAM1* in fibroblasts, cardiac microvascular endothelial cells (CMVECs), and macrophages after miR-146b-5p mimic treatment are

represented by the mRNA level **(C)** and protein level **(D-E)**. m, mimic; c, control. **(F)** Representative figures and quantification of data from the Transwell assay using NIH3T3 fibroblasts (n=5 per group). Lentivirus-mediated *IRAK1* upregulation rescued miR-146b-induced migration. Bar=200 μm. **(G)** ELISA of macrophage-secreted inflammatory factors (n=5 per group). Overexpression of miR-146b-5p reduced inflammatory factor secretion, and overexpression of *IRAK1* rescued these phenotypic changes. **(H)** Representative images and quantification of CMVEC survival under starvation (n=7 per group). Bar=100 μm. **(I)** Tube-formation assay, with a representative image on the left and quantification data on the right (n=3 samples per group, 5 random fields per samples, 3 replicates). Bar=200 μm. Data are expressed as the mean ± SD. *, P<0.05; **, P<0.01. Data were analyzed using one-way ANOVA followed by Tukey's post-hoc analysis. FB, fibroblast; EC, endothelial cell; Mø, macrophage; Lent, Lentivirus; Lent-Ctrl, Lentivirus control; IL, interleukin; TNF, tumor necrosis factor; MCP, monocyte chemotactic protein.

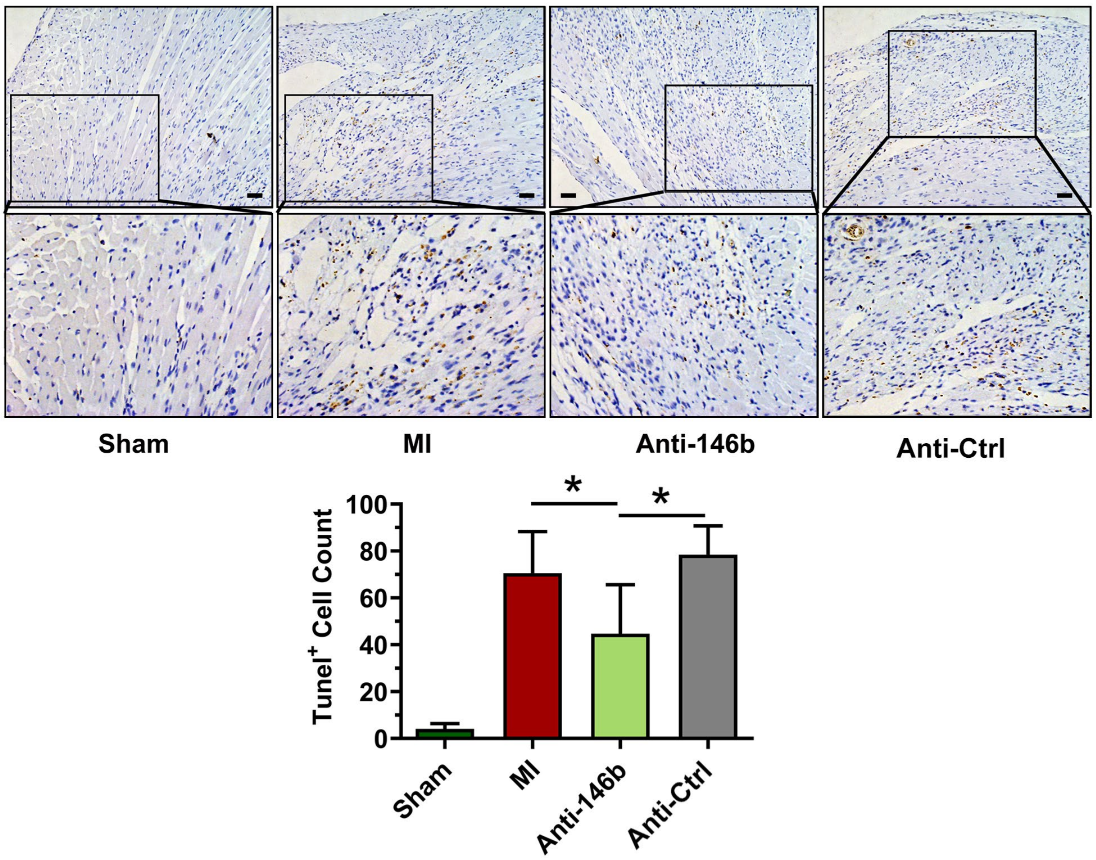

Figure S5. Inhibition of miR-146b-5p alleviated cell apoptosis in the ischemic myocardium

Representative images of TUNEL staining showing that the group subjected to miR-146b-5p inhibition (Anti-146b group) showed significantly lower cell apoptosis than the MI group and antagomir control group (Anti-Ctrl). Blue signals indicate cell nuclei. Brown signals represent TUNEL-positive apoptotic cells (n=7 per group, 10 random fields per sample). Bar=100 μm. Data are expressed as the mean ± SD. *, $P<0.05$. Data were analyzed using one-way ANOVA flowed by Tukey's post-hoc test.

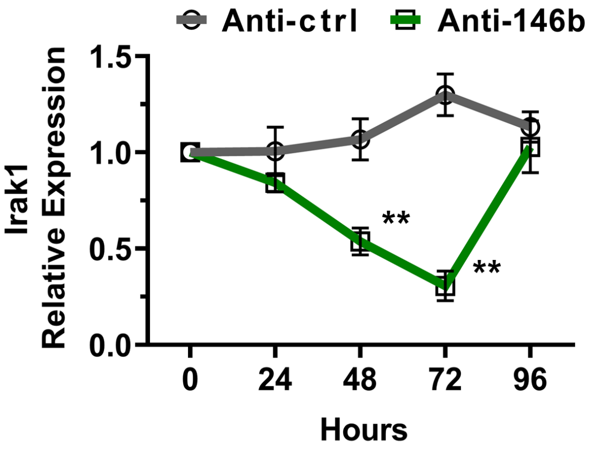

Figure S6. Antagomir efficacy in mouse myocardium

The target gene levels of miR-146b-5p in mouse myocardium were studied to evaluate the efficacy of the miR-146b antagomir in vivo (n=3/group for each time point). Data are expressed as the mean ± SD. **, P<0.01 vs Anti-ctrl group. Data were analyzed using two-way ANOVA flowed by Tukey's post-hoc test. Anti-146b, antagomir miR-146b-5p; Anti-Ctrl, antagomir control.

Supplementary Table

Table S1

Patient characteristic	Coronary normal group (Normal)	Chronic total occlusion group (CTO)
**Demographics**		
Male, no.(%)	3(37.5%)	6 (75%)
Age (years)	58.4±17.0	56.6±7.9
**Clinical parameters**		
Heart rate, per minute	82.5±7.2	78.4±16.6
SBP,mmHg	130.8±17.0	121.6±25.4
DBP,mmHg	79.0±10.8	75.5±13.1
**Comorbidities**		
Hypertension, no.(%)	1（12.5%）	5 (62.5%)
Diabetes mellitus, no.(%)	NA	2 (25%)
Hyperlipidemia, no.(%)	NA	3 (37.5%)
Chronic kidney disease, no.(%)	NA	1 (12.5%)
**Medications**		
Aspirin, no.(%)	NA	5 (62.5%)
Statin, no.(%)	NA	3 (37.5%)
ACEI/ARB, no.(%)	NA	3 (37.5%)
Beta blockers, no.(%)	NA	5 (62.5%)
Clopidogrel, no.(%)	NA	2 (25%)
Cilostazol, no.(%)	NA	1 (12.5%)
Other antihypertensive medications, no.(%)	1(12.5%)	2 (25%)
Other antithrombotic medications, no.(%)	NA	1 (12.5%)
**Laboratory findings**		
Total cholesterol, mM/L	5.1±1.1	3.3±1.0
Triglycerides, mM/L	1.0±0.3	1.6±0.8
HDL cholesterol, mM/L	2.0±0.5	1.0±0.3
LDL cholesterol, mM/L	3.2±0.8	15.0±1.1
Fasting plasma glucose, mM/L	5.2±0.3	6.1±0.9
Glycated hemoglobin, %	NA	5.9±1.0
cTnT, ng/ml	NA	0.0094±0.0044
NT-proBNP, pg/ml	NA	78.3±35.0
Homocysteine, μmol/L	NA	17.6±12.6
Creatine kinase,U/L	NA	121.3±79.6
CK-MB, U/L	NA	21.1±5.4

Table S2

Gene	Forward Primer	Reverse Primer
COL1A1	TAGGCCATTGTGTATGCAGC	ACATGTTCAGCTTTGTGGACC
ACTA2	CAGGCATGGATGGCATCAATCAC	ACTCTAGCTGTGAAGTCAGTGTCG
IRAK1	GAGACCCTTGCTGGTCAGAG	GCTACACCCACCCACAGAGT
CEACAM1	TACATGAAATYGCACAGTCGC	CTGCCCCTGGCGCTTGGAA
GAPDH	CGTGCCGCCTGGAGAAACC	TGGAAGAGTGGGAGTTGCTGTTG
